# Floristic Inventory and Diversity of Urban Green Spaces in the Municipality of Assemini (Sardinia, Italy)

**DOI:** 10.3390/plants14071102

**Published:** 2025-04-02

**Authors:** Marco Sarigu, Lina Podda, Giacomo Calvia, Andrea Lallai, Gianluigi Bacchetta

**Affiliations:** 1 Center for Conservation of Biodiversity (CCB), Department of Life and Environmental Sciences, University of Cagliari, Viale Sant’Ignazio da Laconi, 13, 09123 Cagliari, Italy; msarigu@unica.it (M.S.); andrea.lallai@unica.it (A.L.); bacchet@unica.it (G.B.); 2 Faculty of Agricultural, Environmental and Food Sciences, Free University of Bozen-Bolzano, Piazza Università, 5, 39100 Bozen-Bolzano, Italy; gcalvia@unibz.it

**Keywords:** biodiversity, green cities, invasive species, inventory ornamental plants, public green, sustainability

## Abstract

Urban greenery is a key component of green infrastructure, contributing to environmental sustainability and urban well-being. Between 2019 and 2020, a comprehensive inventory of ornamental flora was conducted in Assemini (Sardinia, Italy), documenting 198 vascular plant taxa, including 155 exotic, 41 native, and 2 cryptogenic species from 65 families. Among the exotic species, most were neophytes (63%), and 14% were archaeophytes. In terms of life forms, scapose phanerophytes, with a tree-like growth habit, dominated (45%), while Mediterranean and American chorotypes were the most represented, each accounting for 21%. A total of 7356 plants were recorded, comprising trees (61.3%), shrubs (32.3%), and climbers (5.7%), belonging to 90 shrub, 89 tree, and 19 climber taxa. The highest number of plants was found in “Green Areas” and “Schools”, which also exhibited the greatest biodiversity, with 136 different taxa each. The most planted species were *Quercus ilex*, *Nerium oleander*, and *Olea europaea*. The survey also identified 21 allergenic, 36 toxic, and 35 mechanically harmful species, primarily located in “Green Areas” and “Schools”. Biodiversity analysis using the Shannon Index revealed significant diversity, with Fabaceae, Apocynaceae, and Fagaceae emerging as the most represented families. These findings highlight the importance of plant inventories in urban green space management for sustainable planning. Well-maintained green spaces can enhance ecological resilience, improve public health, and promote social cohesion in future urban developments.

## 1. Introduction

Urban greenery is broadly categorized under the term “urban forests”. According to the Food and Agriculture Organization (FAO) guidelines, urban forests are classified into five distinct types based on varying degrees of tree cover: peri-urban woodlands, urban parks and forests, small neighborhood parks, private gardens, and green spaces such as street trees, squares, avenues, and other green areas where trees are present. Additionally, natural ecosystems, including tree formations, shrubs, bushes, and wetlands, are also encompassed within this definition [1].

The Italian Strategy for Urban Greenery expands this definition by incorporating a peri-urban forest belt, which serves as a transitional zone between urban areas and natural woodlands within agricultural and natural territorial mosaics. This peri-urban belt is crucial for green infrastructure, enhancing ecological connectivity between natural ecosystems and urban environments [2].

Mehhdi et al. [3] define the concept of “urban green” as spaces designated for accommodating and organizing recreational activities for urban residents. These areas integrate cultural, sports, recreational, and leisure facilities. Furthermore, the Italian National Institute of Statistics [4] specifies that urban green spaces include publicly managed areas such as parks, reserves, and protected areas, which comprise Sites of Community Importance (SCI), Special Areas of Conservation (SAC), and Special Protection Areas (SPA) within municipal territories. These areas, even when managed by non-municipal public bodies and located outside city centers, are recognized as part of urban green spaces.

In recent years, in alignment with the European Green Deal, the EU Biodiversity Strategy for 2030 [5], and European guidelines on biodiversity-friendly afforestation, reforestation, and tree planting [6], the role of urban greenery has been further supported, especially regarding urban forests. Urban forests provide essential ecosystem services such as air pollution reduction, climate change mitigation, and biodiversity conservation [7]. They also offer socio-economic benefits, including aesthetic enhancement and energy savings. However, the development and maintenance of urban green spaces in Italy, particularly in central and southern regions, have lagged behind other European nations and Northern Italian cities [2,8].

Urban green spaces serve multiple functions beyond aesthetics, including acting as windbreaks, acoustic and visual barriers, temperature regulators, and air purifiers. They help mitigate dust, pollutants, and soil erosion caused by water and wind. Recent studies have shown a correlation between the lack of green spaces and increased stress, mental health issues, and vandalism in urban populations [9,10,11]. Despite these benefits, urban green spaces in Italy often remain a low priority for public institutions, resulting in inadequate quality and quantity. According to Italian Ministerial Decree No. 1444 (1968), a minimum of 9 m^2^ of public green space per inhabitant (excluding street trees) and 15 m^2^ for urban and territorial public parks are required. However, few Italian cities meet these urban planning standards, especially when street trees and peri-urban forests are excluded. In contrast, public green space standards in other European countries range from 20 to 25 m^2^ per inhabitant, with some Northern European cities exceeding 100 m^2^ [5].

In recent years, several studies have conducted inventories of urban flora and vegetation in European and non-European cities [12,13,14,15,16,17]. While urban trees and shrubs are often planted for aesthetic purposes, poor planning regarding their growth, ecological requirements, and maintenance often leads to health problems. Factors such as species age, space constraints, soil conditions, root asphyxia, improper pruning, vehicle damage, pollution, and neighborhood socioeconomic status all contribute to these issues [18,19,20]. Moreover, the use of exotic species introduced for aesthetic purposes and trends in the nursery industry are often unchecked, contributing to biological invasions. The deliberate introduction of ornamental plants is recognized as a major driver of plant invasions globally [21], with cities serving as key hubs for their importation and cultivation [22]. Plant invasions endanger biodiversity and natural resources, resulting in economic losses and health concerns such as allergic reactions to pollen and exposure to toxic compounds [23,24]. Prioritizing native species adapted to local bioclimatic conditions is essential, as it prevents resource depletion and protects local biodiversity from the risks of invasion [25].

To support the development and protection of urban green spaces, Law 10/2013, “Norme per lo sviluppo degli spazi urbani” (Regulations for the development of urban spaces), was introduced. This law provides legal tools to assist public administrations in planning and managing urban green spaces, ensuring effective governance and guiding administrators in decision-making processes.

Between 2019 and 2020, an inventory of ornamental tree and shrub taxa in the green spaces of the municipality of Assemini (Metropolitan City of Cagliari, Sardinia) was conducted in collaboration with the Centre for the Conservation of Biodiversity (CCB) at the University of Cagliari.

The main objectives of this study were:

(1). To create, for the first time, a complete inventory of the ornamental flora of Assemini, documenting the diversity of vascular plant taxa, distinguishing between native and exotic species. Additionally, to classify the recorded plant taxa based on their origin and growth forms (trees, shrubs, climbers), as well as analyzing their distribution across different urban green spaces.
(2). To identify the presence of allergenic, toxic, and mechanically harmful plant species, assessing their distribution in public spaces such as parks and schools, in order to reduce health risks to the public.
(3). To use the obtained results to provide the Municipality with a sustainable tool to integrate into the Urban Green Regulation, useful for guiding future urban planning actions and ensuring the sustainable development of green spaces.

## 2. Results

### 2.1. Floristic Composition and Species Richness

The inventory data collected resulted in a comprehensive list of vascular plants, comprising 198 taxa belonging to 65 families. Of these, 41 taxa (21%) were of native origin, 155 (78%) were of exotic origin, and 2 (1%) were cryptogenic. In terms of residence time for the exotic taxa, 27 (14%) were archaeophytes, 125 (63%) were neophytes, and 3 were categorized as horticultural (Table S1).

**Figure 1:**
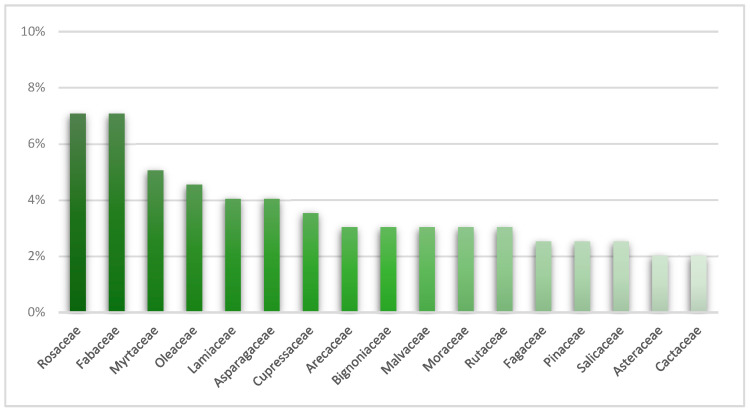
Graphic representation of the percentage distribution of main families of the urban green heritage of the Municipality of Assemini.

The families with the highest biodiversity were Rosaceae (14 taxa) and Fabaceae (13 taxa), followed by Myrtaceae (10 taxa) and Oleaceae (9 taxa), as well as Lamiaceae and Asparagaceae (both with 8 taxa each) (Figure 1). The Rosaceae family exhibited the greatest diversity of genera, with 10 genera, the most prominent of which was *Prunus*.

**Figure 2:**
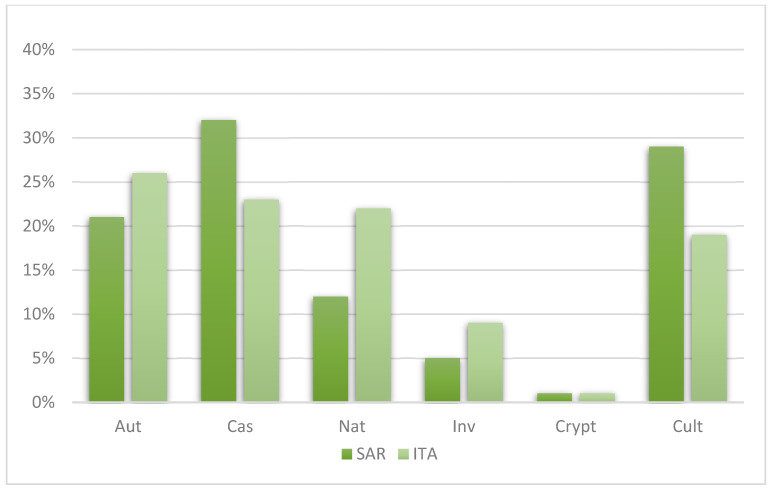
Graphic representation of the percentage distribution by plant status (Aut: autochthonous; Cas: casual; Nat: naturalized; Inv: invasive; Crypt: Cryptogenic; Cult: cultivated) of the ornamental flora of the Municipality of Assemini.

Regarding the status of the 155 exotic taxa, many have been previously recorded as casual species, with 63 taxa identified as casual in Sardinia and 46 in Italy. In contrast, 44 taxa are currently considered naturalized in Italy, compared to 24 in Sardinia (Figure 2). Additionally, there are 10 invasive taxa in Sardinia and 18 at the national level.

**Figure 3:**
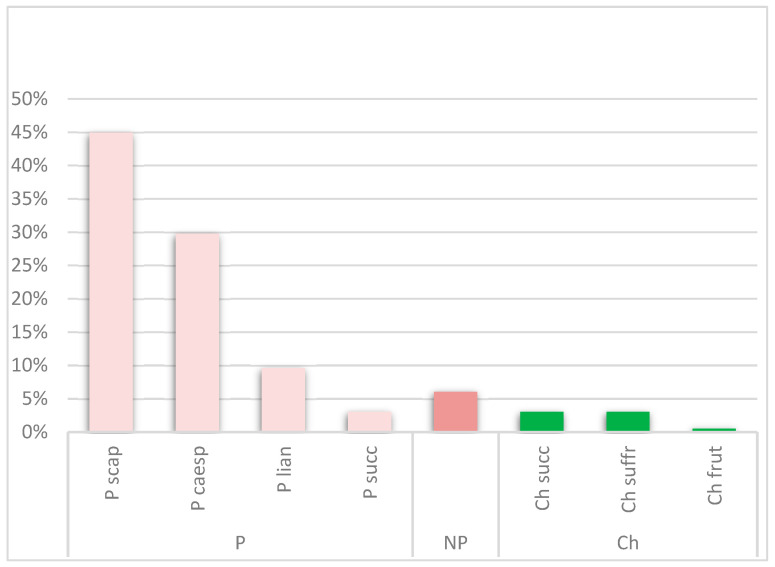
Graphic representation of the percentage distribution by life form (P: phanerophytes; NP: nanophanerophytes; Ch: chamaephytes) and life subform (frut: frutescent; succ: succulent; suffr: suffrutescent; caesp: caespitose; rept: reptant; scap: scapose; lian: liana) of the ornamental flora in the Municipality of Assemini.

The life form analysis revealed a predominance of phanerophytes, with 173 taxa, followed by chamaephytes (13 taxa) and nanophanerophytes (12 taxa). A more detailed analysis of sub-life forms indicated that scapose phanerophytes were the most represented, with 89 taxa, followed by caespitose phanerophytes (59 taxa) and liana phanerophytes (19 taxa) (Figure 3).

**Figure 4:**
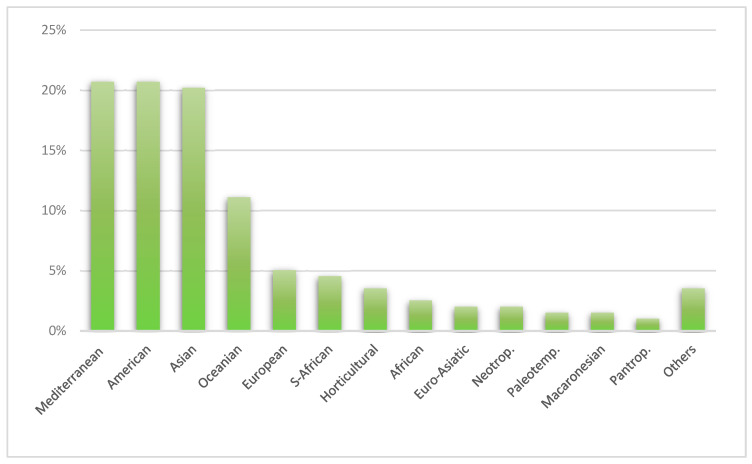
Graphic representation of the percentage distribution of principal chorological data for ornamental taxa in the Municipality of Assemini.

In terms of chorology, the primary sources of taxa for the Municipality of Assemini were Mediterranean and American chorotypes, each contributing 41 taxa, followed by Asian chorotypes with 40 taxa. Other notable contributions included Oceanian (22 taxa), European (10 taxa), and South African (9 taxa) chorotypes (Figure 4).

### 2.2. Inventory Data

Habitus analysis, based on life forms, revealed that out of the total taxa surveyed, 89 (45%) were trees, 90 (45%) were shrubs, and 19 (10%) were climbers (Table S1). Several species exhibited variable growth habits, including *Olea europaea* and *Nerium oleander*, which grew both as trees and shrubs, and *Plumbago auriculata*, which was found as both a shrub and a climber.

**Figure 5:**
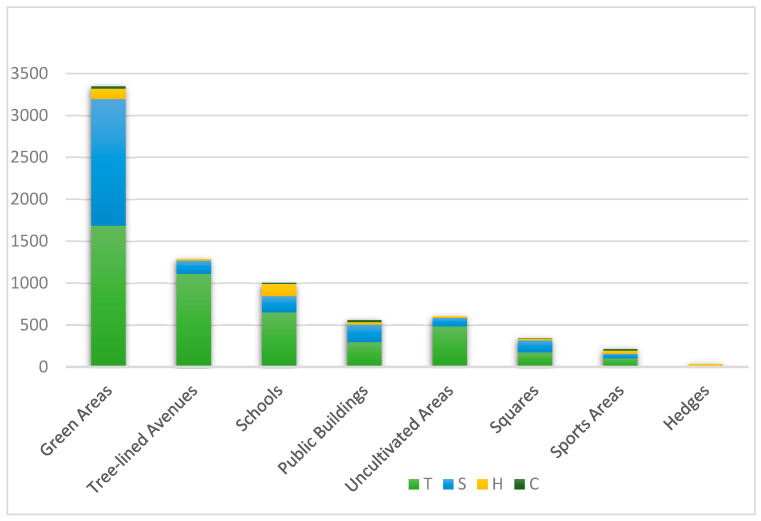
Graphic representation of the number of plants recorded in urban greenery, categorized by habit (T = trees, S = shrubs, H = edges, C = climbers/lianas).

The analysis of individual plant numbers recorded in the database showed a total of 7356 individual plants in the surveyed areas, consisting of 4509 trees (61.3%), 2379 shrubs (32.3%), and 86 climbers (5.7%). Additionally, 418 hedges (1.1%) were identified, though individual counts for hedges were not always possible (Figure 5).

The “Green Areas” category had the highest number of plants (3348 individuals), followed by “Tree-lined Avenues” (1287 individuals) and “Schools” (965 individuals). The highest biodiversity was observed in the “Green Areas” and “Schools” categories, each containing 136 different taxa, compared to 98 in “Public Buildings” and 79 in “Tree-lined Avenues”.

In the “Green Areas”, trees dominated, with 1685 individuals representing 74 taxa. The most prominent species included *Ceratonia siliqua* (212 individuals), *Quercus ilex* (214), *Olea europaea* (172), and *Populus alba* (147). Among the shrubs, *Nerium oleander* was the most frequent species, with 208 individuals, including 15 individuals exhibiting an arboreal habit. The genus *Rosa* was also significant in this category, contributing 610 individuals.

In the “Tree-lined Avenues”, 1287 plants were recorded, of which 1109 were trees and 159 were shrubs. *Quercus ilex* (135 individuals) was the most common tree species, followed by the arboreal form of *Nerium oleander* (134 individuals), *Jacaranda mimosifolia* (130 individuals), *Robinia pseudoacacia* (88 individuals), and *Pinus pinea* (87 individuals).

In the “Schools” gardens, trees predominated (652 individuals), with the most common species being *Quercus ilex* (84 individuals) and *Pinus pinea* (59 individuals). Among the shrub species, *Phillyrea angustifolia*, *Bougainvillea spectabilis*, and *Pistacia lentiscus* were the most frequent. Hedges were also notable in this category, with 147 hedges and high biodiversity (43 species), primarily composed of shrubs like *Myrtus communis*, *Salvia rosmarinus*, and *Ligustrum lucidum*.

In the gardens and flowerbeds of “Public Buildings”, 561 plants were recorded, representing 98 taxa. The most common tree species was *Cupressus sempervirens* (117 individuals), mainly found in the cemetery area, while *Quercus ilex* was present in six out of nine areas, although it had fewer individuals (50). The most frequent shrub species in this category were *Lantana camara* (33 individuals), *Salvia rosmarinus* (27), and *Callistemon citrinus* (25).

In “Uncultivated Areas”, 604 plants were recorded, including 486 trees, 100 shrubs, and 18 hedges. The dominant tree species in these areas were *Olea europaea* (64 individuals), *Ceratonia siliqua* (53), and *Populus alba* (51). The shrub form of *Olea europaea* was also common, represented by the wild variety, likely growing spontaneously in the surveyed areas. Native species like *Pistacia lentiscus* and formerly cultivated species such as *Ficus carica* and *Prunus amygdalus* were also recorded, highlighting the need for conservation efforts.

In the “Squares” category, 343 plants representing 63 taxa were recorded, including 174 trees, 145 shrubs, 19 hedges, and 5 climbing plants. The most frequent species were *Quercus ilex* (44 individuals), *Schinus molle* (24), and *Olea europaea* (22).

The “Sports Areas” contained 180 plants, with 105 trees from 21 taxa and 51 shrubs from 11 taxa. The most represented trees were ×*Hesperotropsis leylandii* (22 individuals) and *Quercus ilex* (16), while the most common shrubs were *Bougainvillea spectabilis*, *Pittosporum tobira*, and *Salvia rosmarinus*.

The “Hedges” category consisted of 20 hedges located in three specific areas (Corso Africa, Corso America, and the hedge leading to Piazza Maiorana). A total of 418 individuals were recorded in this category, with the most commonly used species being *Nerium oleander*, *Viburnum tinus*, *Pyracantha coccinea*, *Polygala myrtifolia*, *Lantana camara*, *Ligustrum lucidum*, and *Ligustrum ovalifolium*.

### 2.3. Relative Abundance of the Most Common Taxon

**Figure 6:**
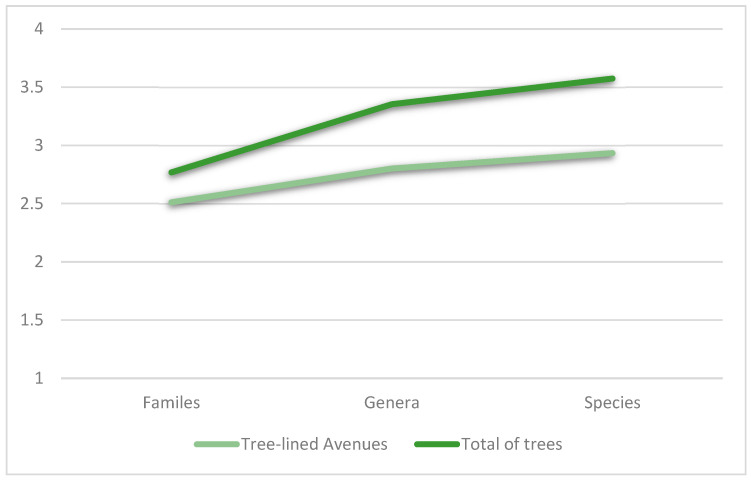
Shannon Index values calculated for the relative abundance of trees, considering families, genera, and species, both for the ’Tree-lined Avenues’ and the total number of trees”.

The diversity values for tree richness, as measured by the Shannon Index, were calculated for both the total number of trees registered across all categories and specifically within the “Tree-lined Avenues” of the Municipality of Assemini. The Shannon Index revealed significant variation, ranging from 2.7 at the family level, 3.3 at the genus level, and 3.5 at the species level for the total trees in “Tree-lined Avenues”. In terms of abundance, the most representative families were Fabaceae (16.2%), Apocynaceae (13.5%), Fagaceae (11.9%), Bignoniaceae (11.5%), and Pinaceae (10.3%). At the genus level, *Nerium* represented 13.5%, followed by *Quercus* (11.9%), *Jacaranda* (11.5%), and *Pinus* (10.3%). Among the species, the most common were *Quercus ilex* (11.3%) and *Nerium oleander* (8.1%) (Figure 6).

### 2.4. Data on Potentially Harmful Plants

Regarding the inventory of potentially harmful plants, the results show the presence of 21 allergenic plants (A) (10.6%), 36 toxic plants (T) (18.2%), and 35 plants causing mechanical injuries (MI) (17.7%).

**Figure 7:**
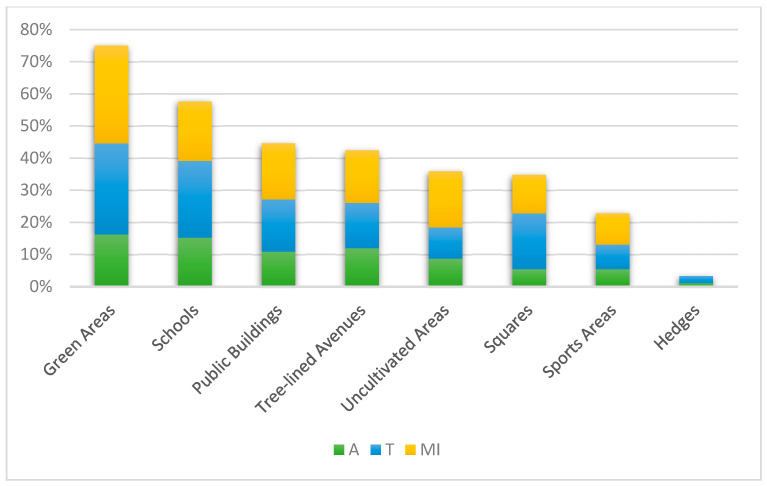
Graphic representation of the number of plants recorded in urban greenery, categorized by potentially dangerous plants (A = allergenic, T = toxic, MI = mechanical injuries).

These plants are primarily distributed across the “Green Areas” category, which contains 69 different taxa, with mechanical injury-causing plants (MI) being predominant (30.4%). Other notable areas include “Schools” with 53 taxa and “Public Buildings” with 44 taxa (Figure 7).

## 3. Discussion

The urban green heritage of the Municipality of Assemini is characterized by remarkable species diversity, comprising a total of 7356 individual plants representing 198 taxa. The prevalence of exotic taxa, particularly neophytes, in urban environments like Assemini highlights the influence of globalization and human intervention, which favor the introduction of ornamental species from various regions, especially from the “New World”. While these species significantly enhance ornamental diversity and are often well-suited to urban conditions, their ecological implications warrant careful consideration. Urban gardens, whether public or private, often serve as points of origin for the escape and proliferation of ornamental plants, which can adversely affect natural habitats [26,27].

Floristic richness, in terms of ornamental plant presence within the urban ecosystem, is represented by 65 families—a noteworthy feature, as over-reliance on a limited number of families can increase vulnerability to pests and diseases. To improve the resilience of the urban ecosystem, plant selection must be diversified, particularly through the inclusion of underrepresented families [28]. The prevalence of plant families such as Rosaceae and Fabaceae in urban areas highlights their ornamental and ecological significance. For example, Fabaceae contribute to nitrogen fixation [29], increasing soil fertility. Their widespread use also aligns with public preferences due to their low maintenance requirements. Similarly, Rosaceae species play a key role in supporting animal diversity by providing food resources, such as berries and fleshy fruits. The Lamiaceae family, composed of several aromatic plants, is also well-represented. Its blooms are particularly valued for attracting key pollinators, including bees and butterflies, thus supporting pollination services vital for ecosystem health [30].

Scapose phanerophytes (trees) and caespitose phanerophytes (shrubs) dominate the urban ornamental flora of Assemini, providing essential ecosystem services such as shade, air purification, and temperature regulation. Trees are also crucial for sheltering vertebrate microfauna and beyond, playing a particularly significant role in supporting avifauna and mammal species. This dominance reflects urban priorities, including efforts to mitigate urban heat islands and improve aesthetic appeal, a trend commonly observed in urban green spaces [31,32,33,34,35]. To promote multifunctional urban landscapes that benefit both people and ecosystems, urban green spaces should incorporate a diversity of vegetation layers, including trees and shrubs [36,37,38]. This layered approach enhances ecosystem functionality while improving ecological resilience. Species such as *Olea europaea* and *Nerium oleander*, which can function as both trees and shrubs, exemplify the adaptability required in urban environments. This versatility allows for optimal space usage and enhances functional diversity, particularly in limited urban areas. However, over-reliance on a limited number of adaptable species can result in homogeneity within urban flora, reducing ecological variety and limiting resilience to environmental stresses. To promote biodiversity while maximizing spatial efficiency and functionality, it is essential to encourage the use of a wider range of adaptable species in urban landscapes [39].

The analysis of chorological forms of plant taxa highlights significant diversity in their provenance. While exotic species constitute the majority, the Mediterranean component is notably well-represented, accounting for 21%, which is equivalent to the proportion of American species. This global distribution reflects a prevailing trend, largely driven by nursery practices and garden design trends, to prioritize ornamental species based on aesthetic appeal, commercial demand, and market availability, rather than native species [40]. Native species are often underrepresented due to limited production and availability in local nurseries. To foster greater sustainability in urban green spaces, local governance should take proactive measures to encourage the use of native species. By doing so, nurseries and garden centers can be steered away from inadvertently serving as hubs for the introduction and spread of invasive alien plants, ensuring that urban ecosystems remain both ecologically resilient and environmentally responsible [40].

Regarding the floristic component of exotic origin, it is important to note that most exotic plants recorded in the study area do not exhibit, at the local level, a spontaneous establishment status classified as casual, naturalized, or invasive. Instead, the assigned status is based on classifications determined at the regional (Sardinian) and national (Italian) levels to assess the potential spread and/or invasion of these species. Among the recorded exotic taxa, only eight species (*Acacia saligna*, *Arundo donax*, *Austrocylindropuntia subulata*, *Lycium ferocissimum*, *Opuntia ficus-indica*, *Passiflora morifolia*, *Robinia pseudoacacia*, and *Vachellia karroo*) were observed growing spontaneously beyond garden boundaries, primarily in marginal areas. This suggests their probable escape from cultivation and subsequent diffusion. Six of these species are classified as invasive for Sardinian flora, while *Lycium ferocissimum* and *Passiflora morifolia*, though not officially designated as invasive, are locally problematic and may pose a significant threat to the sensitive ecosystems surrounding extra-urban areas. In particular, *Passiflora morifolia* was recorded for the first time in Sardinia and in Italy in this area [41].

In addition to these invasive and spontaneously spreading species, five other taxa considered invasive in Sardinia (*Agave ingens*, *Eucalyptus camaldulensis* subsp. *camaldulensis*, *Malephora crocea*, *Parkinsonia aculeata*, and *Senecio angulatus*) have been found confined to gardens and uncultivated areas. While these species have not yet shown signs of naturalization at the local level, their potential to become problematic in the future cannot be overlooked. Similarly, the 18 taxa listed as invasive for Italian flora (Table S1) also represent a latent risk. Given these concerns, maintaining a comprehensive inventory of urban green space species is essential for informed management decisions and to prevent the inadvertent use of invasive species as ornamentals, thereby mitigating potential ecological threats [42,43].

Invasive alien species pose significant risks to native flora by outcompeting native species, destabilizing ecosystems, reducing biodiversity, and potentially causing irreversible changes to ecosystem dynamics. However, some alien species, often supported by climate change, demonstrate greater resilience to urban stresses such as pollution, compacted soils, and temperature fluctuations—conditions in which native species often struggle to thrive. Additionally, alien plants can provide economic and aesthetic benefits that native species may not always offer, making them a preferred choice in certain urban contexts. However, to avoid serious threats to local biodiversity, continuous monitoring with targeted eradication efforts (e.g., for *Robinia pseudoacacia* and *Arundo donax*) and well-planned restoration interventions are necessary to prevent ecosystem degradation and biodiversity loss [44,45].

Among the area types analyzed, “Green Areas” and “Schools” present the highest levels of biodiversity, benefiting from larger spaces and multifunctional roles. These areas act as vital reserves of urban biodiversity while providing opportunities for education and recreation. In contrast, “Tree-lined Avenues” and “Squares” present lower biodiversity, mainly due to space limitations and design priorities focused on shading and aesthetic appeal. This reduced diversity can increase vulnerability to environmental stresses.

The analysis of tree diversity in the Municipality of Assemini, particularly within the “Tree-lined Avenues”, highlights considerable variations in species richness and abundance. Shannon Index values indicate a relatively high level of diversity, with values increasing from the family level to the genus and species levels. This pattern suggests that while tree diversity is well-distributed at the species level, some taxonomic groups dominate in terms of abundance. These results were compared with similar studies conducted by Flores et al. [46], who highlighted similar trends in the analysis of tree diversity, confirming the validity of the approach adopted and underlining the importance of local variability in species composition. The prevalence of Fabaceae, Apocynaceae, and Fagaceae at the family level indicates a tendency towards species that are well-adapted to urban conditions. *Nerium oleander*, for example, is widely used in Mediterranean urban landscapes for its drought resistance and ornamental value. Similarly, *Quercus ilex* is a native species known for its resilience in urban and peri-urban environments, contributing both ecological and aesthetic benefits. However, *Quercus ilex* is particularly vulnerable to infestation by *Nidularia pulvinata*, a scale insect that progressively damages holm oaks by extracting sap, leading to crown desiccation over time [47].

From an ecological perspective, the predominance of certain genera, such as *Nerium*, *Quercus*, and *Jacaranda*, suggests that tree selection in urban planning could prioritize aesthetics, drought tolerance, and adaptability. However, over-reliance on a limited number of species could pose risks, particularly in terms of susceptibility to pests and diseases. Lohr et al. [48] emphasize that limited species and genetic diversity in urban trees globally increases the risk of tree loss, particularly in the face of climate change. Commonly planted species, selected for their adaptability and visual appeal, are often overused, resulting in monocultures that are highly susceptible to pests and disease.

The analysis of potentially harmful species in urban green spaces has highlighted a significant concern for public health and safety, especially in areas like “Green Areas” and “Schools”. These spaces host several species that pose risks, such as causing allergies from pollen or contact, toxicity from ingestion, and mechanical injuries. By “mechanical injuries”, we mean physical injuries resulting from direct contact with plants, such as scratches, punctures, or other forms of damage caused by plant structures such as thorns or spines [49]. The risks are particularly pronounced for children, who may unknowingly ingest parts of toxic plants such as *Nerium oleander* and *Cycas revoluta* [50]. The allure of colorful flowers, leaves, and fruits can attract children to these species, making them unsuitable for public spaces frequented by young people [51].

To mitigate the rise of pollen allergies, urban green space design should prioritize native species with low allergenic pollen, favoring sterile or female plants and entomophilous species. Furthermore, mechanical injury risks must be carefully considered, with attention given to species like *Robinia pseudoacacia*, *Agave* sp. pl., and thorny plants of the genus *Bougainvillea* or *Rosa*.

Urban greenery management and planning can be implemented through various strategies that integrate the findings of the urban flora study.

First and foremost, it is essential to adopt a biodiversity-based planning approach, using species diversity data obtained from the inventory to design balanced and resilient green spaces. It is crucial to prioritize the use of native species to strengthen ecological resilience and reduce maintenance needs while also creating diversified habitats to support local wildlife.

Urban greenery must be integrated into all stages of urban planning, harmonizing it with buildings, streets, and public spaces. It is important to promote green infrastructure, create new green areas in densely populated neighborhoods, and improve park accessibility, ensuring an equitable distribution of green spaces.

Another key aspect is sustainable maintenance, which can be achieved by adopting environmentally low-impact management practices, such as efficient irrigation and reducing pesticide use. Additionally, it is necessary to monitor the growth and spread of invasive species that could disrupt ecological balance.

Finally, it is essential to mitigate environmental and health risks by reducing the presence of allergenic and toxic species in sensitive areas, such as schools, and using greenery to reduce air pollution and urban noise. Green spaces can also help counteract the urban heat island effect by choosing species suited to the local microclimate in light of ongoing climate changes.

To ensure the success of these strategies, the involvement of the local community is fundamental, promoting biodiversity awareness programs and actively engaging citizens in urban greenery management decisions, thus encouraging greater participation and a collective responsibility.

## 4. Materials and Methods

### 4.1. The Study Area

Assemini (39°17′13.14″ N 9°00′19.37″ E) is a municipality with a population of 25,654, located in the Metropolitan City of Cagliari (Southern Sardinia, Italy). It spans an area of 118.17 km^2^ and is situated approximately 8 km northwest of Cagliari. Assemini has been continuously inhabited since prehistoric times, from the Bronze Age to the beginning of the Iron Age (1600–510 BC), with significant development occurring between the 10th and 11th centuries [52].

According to weather data from the nearby Elmas and Decimomannu airports, the average minimum temperature in the coldest month is 5.5 °C, with an average of 3.1 frost days (≤0 °C), and absolute minimum temperatures do not fall below −5 °C. During summer, the average maximum temperature is 30 °C, with an average of 26.7 hot days (≥30 °C), and absolute maximum temperatures do not exceed 46.8 °C. Precipitation averages 483.5 mm per year, concentrated from autumn to spring, with a marked summer drought [53]. Bioclimatically, Assemini falls within the Mediterranean Pluviseasonal Oceanic bioclimate, classified within the Thermo-Mediterranean superior/Upper dry Mediterranean zone [54].

The urban green area covers approximately 170,000 m^2^, with potential vegetation classified as edafo-hygrophilous and lowland geosigmetum (*Populenion albae*, *Fraxino angustifoliae-Ulmenion minoris*, *Salicion albae*) [55].

The landscape surrounding Assemini is primarily characterized by plains, predominantly composed of Quaternary alluvial deposits, with smaller sections of fluvial-lacustrine and marine-lagoon formations. These deposits are the result of detritus transported by key streams and rivers, including Rio S. Lucia to the west, Rio de Giaccu Meloni, Rio Sa Murta, and Rio di Sestu to the east, and Rio Flumini Mannu and Rio Cixerri to the west and south. The municipality also includes the Gutturu Mannu administrative island, a 5000-hectare area that features the abandoned S. Leone mine and the Santa Gilla Lagoon. Although specific studies have been conducted on the flora and vegetation of the Santa Gilla Lagoon and the Monte Arcosu Nature Reserve [56,57], a comprehensive survey of the urban green areas in Assemini had not been carried out or published prior to this project.

**Figure 8:**
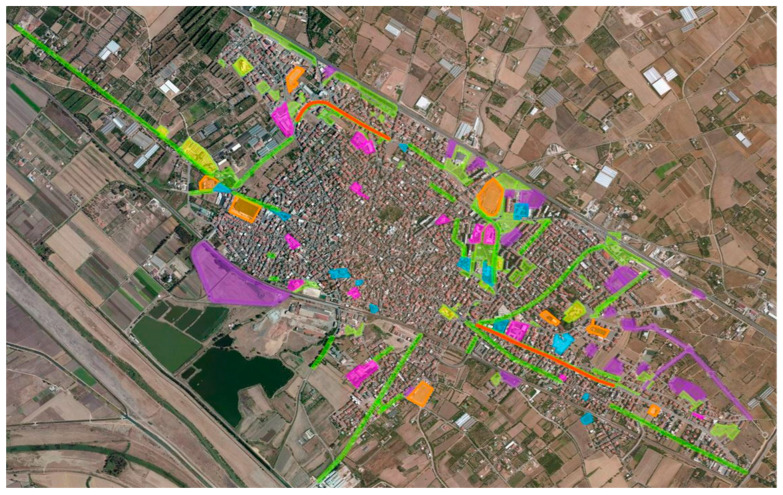
Georeferenced map of green areas. Green: Green Areas; Light Green: Tree-lined Avenues and Hedges; Violet: Uncultivated Areas; Blue = Squares; Pink: Schools; Yellow: Public Buildings; Orange: Sports Areas.

We conducted the field surveys for this project in different sections of the municipality, categorized into eight typologies: Green Areas, Tree-lined Avenues, Uncultivated Areas, Squares, Schools, Public Buildings, Sports Areas, and Hedges. This classification was based on geospatial information provided by the Municipality of Assemini, accessible through the geonue platform at the following link: https://umap.geonue.com/it/map/mappa-aree-verdi_1171#15/39.2950/9.0110 (accessed on 28 February 2025) (Figure 8).

### 4.2. Data Collection

We conducted the inventory work through field trips aimed at quantifying the exact heritage of ornamental plants cultivated by the Municipality and present throughout its territory. This data were provided to the technical office to monitor the presence/absence of plants over time, as well as to verify their phytosanitary status. The comprehensive inventory was carried out through systematic field surveys, with data meticulously documented on standardized survey forms and subsequently digitized into an .xls database format. The inventory was organized alphabetically, starting from the family level, followed by genus and scientific name. We also reported life forms and chorology, specifying whether each species was native (autochthonous) or exotic (non-native, alien, allochthonous). For exotic species, we provided additional details on their introduction period (residence time), categorizing them as archaeophytes (introduced before 1492) or neophytes (introduced after 1492), and horticultural species, as well as their status in Sardinia and Italy. The status was classified as casual, naturalized, invasive, or cultivated, according to Galasso et al. [58] and subsequent updates (Table S1). Additionally, we included information about cryptogenic plants [59].

To ensure precise species taxonomic identification, various national and international botanical references were consulted, including Pignatti et al. [60], Arrigoni [61], and Brickell [62], alongside herbarium specimens from the Herbarium CAG (University of Cagliari Herbarium) and online resources such as Arboles ornamentales [63] and the Royal Horticultural Society [64]. For plant families, we followed the Angiosperm Phylogeny Group IV [65]. In our study, we define a “taxon” following the international botanical taxonomic nomenclature, which includes each taxonomic rank, both supra-specific (genus, family) and infra-specific (species, subspecies, variety). The status of exotic species and taxonomic nomenclature adhered to the Checklist of the Native and Alien Flora of Italy [58,66] and was cross-referenced with online databases such as WFO Plant [67], POWO [68], and Med-Checklist [69]. Life forms were expressed according to Raunkiaer’s classification system [70], using the abbreviations reported in Pignatti et al. [60] and IPFI [71]. The chorology of plants was based on POWO [68], IPFI [71], or relevant literature. The habitus of plants was classified according to Raunkiaer’s life forms [70], including in trees: scapose phanerophytes (P scap); in shrubs: caespitose phanerophytes (P caesp), nanophanerophytes (NP), frutescent chamaephytes (Ch frut), suffruticose chamaephytes (Ch suffr), and succulent chamaephytes (Ch succ); in climbers: liana phanerophytes (P lian).

Potentially harmful plants were classified and evaluated according to Nelson et al. [72] and Appendino et Ballero [73] into the following categories:

- Allergenic plants—Species that can trigger allergic reactions, including respiratory, skin, or systemic hypersensitivity responses.
- Toxic plants—Plants containing toxic compounds that may cause poisoning through ingestion, skin contact, or inhalation.
- Plants causing mechanical injuries—Species with structural features such as thorns, spines, or abrasive surfaces capable of causing cuts, punctures, or irritation.

### 4.3. Measuring Biodiversity

To assess species biodiversity, the Shannon Index, a widely used metric for quantifying diversity by measuring the uncertainty in identifying a random individual, was calculated. The Shannon Index typically ranges from 1.5 (low diversity) to 3.5 (high diversity), and values rarely exceed 4.5 [74]. Calculations of the Shannon Index were performed at the species, genus, and family levels using the Past 4.13 statistical software. Simple linear regressions were then applied to test whether the relative abundance of the most common taxa could predict diversity, using log-transformed relative abundance values to meet the assumptions of the regression analysis.

The Shannon Index (or Shannon–Wiener Diversity Index) is a popular metric in ecology. Based on Claude Shannon’s formula for entropy, it estimates the diversity of species in a community. The index takes into account both the number of species (richness) and their relative abundance (evenness).

The Shannon Index reflects diversity within the taxa of a specific community. Its values increase with both the number of species and the uniformity of their abundance.

## 5. Conclusions

The study of the urban green of Assemini reveals significant plant heritage, with 7356 individual plants from 198 different taxa. The high percentage of exotic species confirms the impact of globalization; in particular, the use of ornamental species that are fashionable in the world of green designers. While these plants enhance ornamental diversity, their ecological impact necessitates careful management to prevent the spread of invasive species into natural habitats. In fact, as many as eight species have escaped beyond the boundaries of gardens and could threaten the stability of ecosystems, particularly invasive species such as *Robinia pseudoacacia* and *Arundo donax*. Effective solutions require continuous monitoring such as eradication of invasive species and prioritization of native species. Climate change intensifies these challenges, increasing drought stress and altering species distributions. While drought-tolerant species are essential, overuse risks ecological homogenization. On the contrary, the percentage of native species in urban green areas is not competitive enough, and their use should be encouraged for the numerous benefits on health and the environment.

This study identifies “Green Areas” and “Schools” as key biodiversity reserves, benefiting from space and multifunctionality, while they are also the areas most affected by harmful plants. The potential health risks posed by toxic or allergenic species emphasize the need for careful plant selection in areas frequented by children.

In conclusion, this study advocates for a biodiversity-based approach to urban green space planning, emphasizing the importance of native species, diversifying plant species, and managing invasive and harmful species to create resilient urban ecosystems.

## Data Availability

The original contributions presented in this study are included in the article/Supplementary Material. Further inquiries can be directed to the corresponding author.

## Supplementary Materials

The following supporting information can be downloaded at: https://www.mdpi.com/article/10.3390/plants14071102/s1, Table S1: Inventory of urban greenery of Assemini. Life form (P = phanerophytes, NP = nanophanerophytes, Ch = chamaephytes); life-subform (frut: frutescent, succ: succulent, suffr: suffrutescent, caesp: caespitose, scap: scapose, lian: lianas).
